# Amyloid-β and Proinflammatory Cytokines Utilize a Prion Protein-Dependent Pathway to Activate NADPH Oxidase and Induce Cofilin-Actin Rods in Hippocampal Neurons

**DOI:** 10.1371/journal.pone.0095995

**Published:** 2014-04-23

**Authors:** Keifer P. Walsh, Laurie S. Minamide, Sarah J. Kane, Alisa E. Shaw, David R. Brown, Bruce Pulford, Mark D. Zabel, J. David Lambeth, Thomas B. Kuhn, James R. Bamburg

**Affiliations:** 1 Department of Biochemistry and Molecular Biology, Colorado State University, Fort Collins, Colorado, United States of America; 2 Molecular, Cellular and Integrative Neuroscience Program, Colorado State University, Fort Collins, Colorado, United States of America; 3 Department of Biology and Biochemistry, University of Bath, Bath, United Kingdom; 4 Prion Research Center, Department of Microbiology, Immunology and Pathology, Colorado State University, Fort Collins, Colorado, United States of America; 5 Department of Pathology, Emory University School of Medicine, Atlanta, Georgia, United States of America; 6 Department of Chemistry and Biochemistry, University of Alaska, Fairbanks, Alaska, United States of America; University of Maryland School of Medicine, United States of America

## Abstract

Neurites of neurons under acute or chronic stress form bundles of filaments (rods) containing 1∶1 cofilin∶actin, which impair transport and synaptic function. Rods contain disulfide cross-linked cofilin and are induced by treatments resulting in oxidative stress. Rods form rapidly (5–30 min) in >80% of cultured hippocampal or cortical neurons treated with excitotoxic levels of glutamate or energy depleted (hypoxia/ischemia or mitochondrial inhibitors). In contrast, slow rod formation (50% of maximum response in ∼6 h) occurs in a subpopulation (∼20%) of hippocampal neurons upon exposure to soluble human amyloid-β dimer/trimer (Aβd/t) at subnanomolar concentrations. Here we show that proinflammatory cytokines (TNFα, IL-1β, IL-6) also induce rods at the same rate and within the same neuronal population as Aβd/t. Neurons from prion (PrP^C^)-null mice form rods in response to glutamate or antimycin A, but not in response to proinflammatory cytokines or Aβd/t. Two pathways inducing rod formation were confirmed by demonstrating that NADPH-oxidase (NOX) activity is required for prion-dependent rod formation, but not for rods induced by glutamate or energy depletion. Surprisingly, overexpression of PrP^C^ is by itself sufficient to induce rods in over 40% of hippocampal neurons through the NOX-dependent pathway. Persistence of PrP^C^-dependent rods requires the continuous activity of NOX. Removing inducers or inhibiting NOX activity in cells containing PrP^C^-dependent rods causes rod disappearance with a half-life of about 36 min. Cofilin-actin rods provide a mechanism for synapse loss bridging the amyloid and cytokine hypotheses for Alzheimer disease, and may explain how functionally diverse Aβ-binding membrane proteins induce synaptic dysfunction.

## Introduction

Bundles of 1∶1 actin∶cofilin (cofilin-actin rods) impair synaptic function and are found in Alzheimer disease (AD) brain [1], [2] and in brains of aged rats [3]. Rods are induced in neurites by stimuli that increase dephosphorylated (active) cofilin, including treatment with β-amyloid (Aβ) peptides, major initiators of Alzheimer disease [1], [4]–[7]. Rods may mediate synaptic loss induced by Aβ [5]-[7], by either sequestering cofilin from dendritic spines where it functions in long-term potentiation (LTP) [8] or blocking vesicle transport [3], [4], [9].

Notably, oxidation of synthetic human Aβ_1-42_ to generate SDS-stable dimers increased its rod-inducing activity by 600 fold [7]; SDS-stable dimers are a major form of Aβ in the soluble pool extracted from most AD brains [10], [11]. Aβ binds promiscuously to different partners found in postsynaptic termini [12]. Each Aβ-binding partner can influence cofilin phosphoregulation as well as synaptic function [13]–[15]. In addition to cofilin dephosphorylation, rod formation *in vivo* requires production of reactive oxygen species (ROS) to generate disulfide-linked cofilin dimers [16]. Because increased oxidative stress markers are found in brains of amnestic mild cognitively impaired (aMCI) subjects most of whom are pre-AD [17], oxidation of Aβ and cofilin may be contemporaneous.

Proinflammatory cytokines, e.g. TNFα, may initiate and enhance the oxidative cascade of neurodegeneration [18], [19]. TNFα stimulates NADPH oxidase (NOX) and ROS production in many cell types including neurons [20]. NOX activity in humans is inversely correlated with cognition [21]. In a mouse model for AD, enhanced NOX activity was linked to cognitive impairment [22], whereas NOX inhibition had beneficial effects [23].

One Aβ binding partner, cellular prion protein (PrP^C^) [24], acts as a co-receptor with other membrane proteins, such as neuronal cell adhesion molecule (NCAM) [25] or the metabotropic glutamate receptor mGluR5 [26], to activate fyn tyrosine kinase, a signaling pathway implicated in neurite outgrowth and synaptic dysfunction [27], [28]. Aβ-mediated inhibition of long-term potentiation (LTP) [29] and Aβ-induced cognitive deficits in an AD mouse model [30] are prevented by blocking Aβ-PrP^C^ interaction, thus implicating this interaction in Aβ-mediated synaptic impairment. However, the molecular mechanism(s) by which impairment occurs is unknown.

Here we report that cofilin-actin rods are induced by proinflammatory cytokines and Aβ in the identical subpopulation of hippocampal neurons. Rod formation induced by Aβ and TNFα, but not by glutamate or mitochondrial inhibitors, utilizes a PrP^C^-dependent pathway activating NOX. PrP^C^ overexpression is sufficient to induce rods in a NOX-dependent manner suggesting a common mechanism by which multiple and functionally diverse Aβ-binding membrane proteins might cause synaptic dysfunction.

## Results

### Proinflammatory cytokines have rod-inducing ability

**Figure 1:**
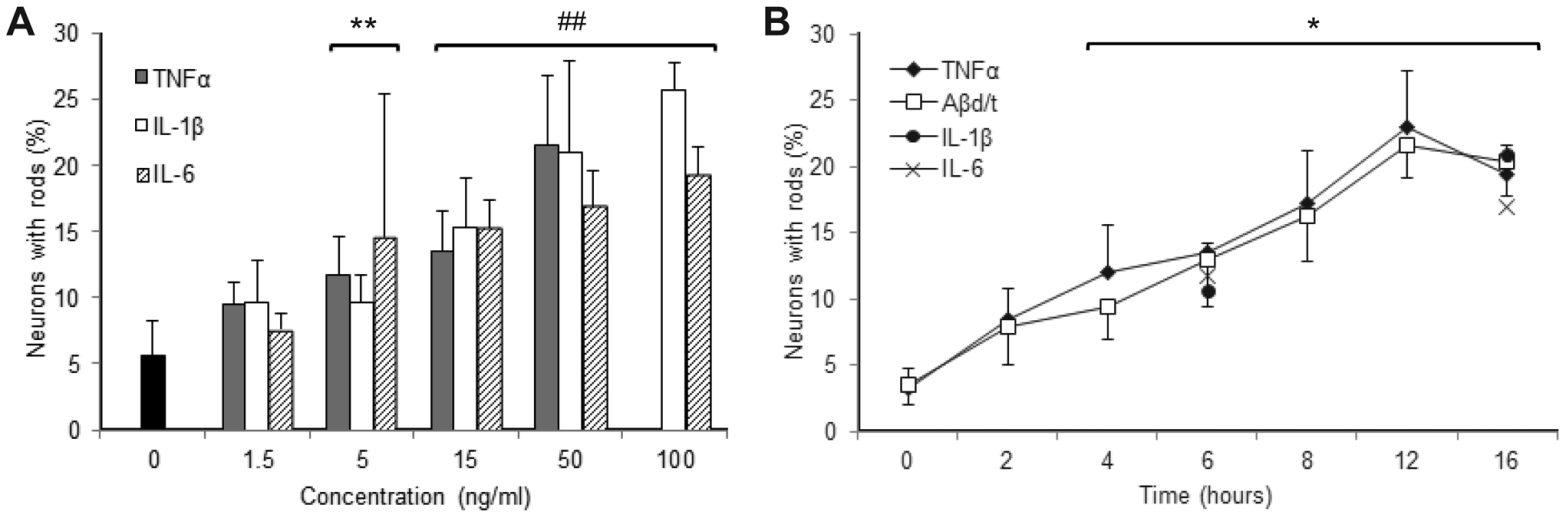
Proinflammatory cytokine dose-response curves and time course of rod formation in dissociated hippocampal neurons. (A) Percent of neurons with rods at 20 hr after treatment with TNFα, IL-1β and IL-6 show a similar dose-response. The maximum response level of approximately 20% of the neurons was reached at ∼50 ng/ml for each cytokine. Higher doses of TNFα kill the neurons within 12–24 hr, so 50 ng/ml was selected for further studies on treatments of up to 24 h. (B) The time course of rod formation in dissociated hippocampal neurons treated with 50 ng/ml TNFα is remarkably similar to that for Aβd/t which is used at ∼250 pM concentration [7]. Six and 16 h time points were performed with IL-1β and IL6 and they are not significantly different from TNFα and Aβ responses at the same times. Significance values with respect to untreated or zero time controls: *(p<0.01), **(p<0.05), ## (p<0.005). Error bars in this and all subsequent figures are standard deviations.

Cofilin oxidation is a prerequisite for the formation of cofilin-actin rods in hippocampal neurons [16]. Since proinflammatory cytokines initiate oxidative stress in neurons [31], we examined whether the major proinflammatory cytokines in the brain, i.e. TNFα, IL-1β and IL-6 [32], elicit rod formation in dissociated rat (E18) hippocampal neuronal cultures. The percent of neurons forming rods as a function of cytokine concentration was quantified. Each of the three cytokines induced rods significantly (p<0.05) above untreated control at 5 ng/ml, and in a maximum of 17–26% of the neurons at a concentration of 50–100 ng/ml (p<0.005) (Figure 1A). The time course of rod formation mediated by the proinflammatory cytokines (Figure 1B) was indistinguishable to that obtained with an optimal concentration of SDS-stable Aβ dimer/trimer (Aβd/t) [7], reaching a significant difference (p<0.01) over controls by 4 h with 50% maximal response reached by 6 h of treatment (Figure 1B). TNFα was selected as the proinflammatory cytokine for further study.

### TNFα and Aβd/t induce rods in the same population of hippocampal neurons

**Figure 2:**
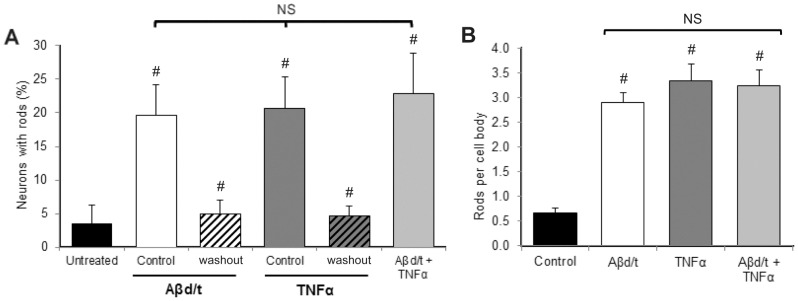
Rod formation in neurons in response to Aβd/t and TNFα used separately or together and their reversibility 24 h after wash out. (A) Percent of neurons forming rods 24 h after treatment with 50 ng/ml TNFα or Aβd/t (∼250 pM) are the same and when used together there is no significant increase in response, strongly suggesting that an identical population of neurons respond to both stimuli. Rods disappear by 24 hr after removal (washout) of the Aβd/t ot TNFα. Treatments compared to the untreated control and washout compared to their treated controls are significant (# p<0.001). Differences in rod response between treatments are not significant (NS). (B) Rod numbers per cell body between treatments are significant (# p<0.001) from untreated control, but are not significant (NS) between the treatments.

The nearly identical time course and maximal rod response in neurons treated with TNFα and Aβd/t suggested that these might be affecting the identical neuronal population. To test this hypothesis, we treated cultures of dissociated rat hippocampal neurons with optimal rod-inducing concentrations of Aβd/t (∼250 pM) or TNFα (2.9 nM = 50 ng/ml) alone and together. After 24 h of treatment, neurons were fixed, immunostained for cofilin, and rods quantified both in terms of the percent of neurons with rods and the number rod index (rods per field or per cell body), which gives an estimate of the robustness of the rod response for each responding neuron. TNFα and Aβd/t induce rods in 20–25% of the neurons (p<0.001 with respect to untreated controls) and neither the population of responding neurons (Figures 1, 2) nor the magnitude of the response (Figure 2B) increased when both rod inducers are used together. This finding suggests that Aβd/t and TNFα induce rods in the same neuronal population.

### Rod induction by TNFα and Aβd/t, but not by glutamate or the mitochondrial inhibitor antimycin A, require the presence of the cellular prion protein

**Figure 3:**
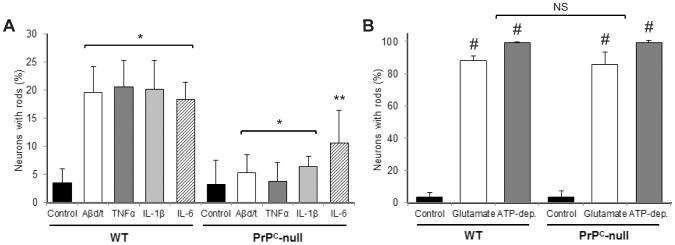
The cellular prion protein, PrP^C^, is required for rod formation from Aβd/t and proinflammatory cytokines, but not from rods induced by glutamate or mitochondrial inhibitors. (A) Percent of neurons with rods 20 h after treatment with Aβd/t or proinflammatory cytokines measured in dissociated neurons from FVB wild type mice or from the PrP^C^-null mouse made in the FVB background. All of the decreases in the response of PrP^C^-null neurons are significant with respect to their wild type controls (* p<0.01; ** p<0.05). (B) Rod formation is significant (# p<0.001) with respect to untreated neurons but does not differ significantly (NS) between hippocampal neurons from wild type (WT) and PrP^C^-null mice in response to excitotoxic levels of glutamate (150 µM) or ATP-depletion (10 mM NaN_3_, 2 mM 2-deoxyglucose) demonstrating that neither of these rod-inducing stresses utilize a PrP^C^-dependent pathway.

PrP^c^ interacts directly with Aβ [24] and is required for Aβ-induced loss of LTP *in vitro* [29] and cognitive deficits in AD mouse models [33]. Thus, if rods are to be considered a plausible mechanism for the synapse dysfunction induced by Aβd/t, then there should be a dependence on PrP^C^ for their formation induced by Aβ. Hence, we cultured hippocampal neurons from PrP^C^-null mice (P0) and compared rod formation in response to Aβd/t (250 pM) and proinflammatory cytokines (each at 50 ng/ml) to hippocampal neurons from P0 wild type (wt) mice of the same line (FVB). Neurons from wt mice showed the typical 20–25% rod response (Figure 3A), whereas rod formation in PrP^C^-null neurons was significantly reduced to that of untreated controls (p<0.01 for Aβd/t, TNFα, and IL-1β; p<0.05 for IL-6). Importantly, the robust and significant (p<0.001) rod response to excitotoxic levels of glutamate or to ATP-depletion (addition of mitochondrial inhibitors with or without the glycolysis inhibitor 2-deoxy-D-glucose) was not dependent on the presence of PrP^C^ (Figure 3B). These findings demonstrate that the hippocampus of both rats and mice have a similar subpopulation of neurons that form rods with the same inducing agents and that there are at least two independent pathways leading to rod formation in hippocampal neurons.

### NADPH oxidase activity is required for cofilin-actin rod formation in the prion protein-dependent pathway

Oxidative stress markers in brain increase during early stages of human cognitive impairment [17] and correlate with enhanced activity of NOX [21]. NOX activation, recognized as a principal source of oxidative stress in many chronic central nervous system (CNS) disorders [23], [34], is stimulated in neurons by TNFα [20]. To first determine that prion knock-out mice were expressing the major NOX isoforms, NOX1 and NOX2, immunoblots were performed which showed no change in their expression in extracts from wt and PrP^C^-null mouse brain (Figure S1).

**Figure 4:**
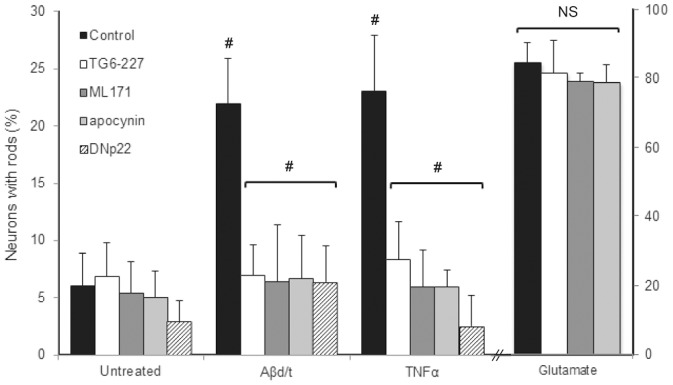
Both Aβd/t and TNFα utilize an NADPH oxidase-dependent pathway for rod formation, whereas glutamate does not. Hippocampal neurons were either untreated or infected with adenovirus for expressing DNp22^PHOX^ for 48 h prior to day 5 in culture. Some cultures were pre-treated 1 h with NOX inhibitorsTG6-227 (1 µM), ML171 (500 nM) or apocynin (1 µM). In the continued presence of the NOX inhibitors (or in neurons expressing DNp22 for 48 h), neurons were treated for 20 h with Aβd/t (∼250 pM) or TNFα (50 ng/ml), or for 30 min with glutamate (150 µM) before fixing, immunostaining for cofilin and quantifying the percent of neurons with rods. Rod response to Aβd/t and TNFα, but not glutamate, was significantly (# p<0.001) reduced by each of the NOX inhibitors.

To test the hypothesis that NOX activity and the subsequent ROS production is required for rod formation in the PrP^C^-dependent pathway, we used both dominant interfering and pharmacological approaches to block NOX, for which isoforms 1, 2 and 4 have been identified in CNS neurons [35]–[37]. The P156Q mutation in the NOX subunit p22^PHOX^ exerts a dominant negative (DN) effect preventing the recruitment of Noxo1/p47^PHOX^ subunits and thus rendering NOX isoforms 1–3 inactive [37], [38]. We generated a replication-deficient, recombinant adenovirus to co-express DNp22^PHOX^ (Ad-DNp22^PHOX^) and green fluorescent protein (GFP) under separate promoters [39]. We tested the ability of the expressed DNp22^PHOX^ to prevent the increase in ROS induced by phorbol myristate acetate (PMA) treatment in the readily infected osteosarcoma SAOS2 cell line using oxidation of 2′,7′dihydrodichlorofluorescein (DCF) as a measure of ROS [31]. Uninfected and virus control (mRFP alone) infected cells responded identically to PMA whereas the increase in ROS due to PMA was inhibited by DNp22^PHOX^ (). The visualization of DCF oxidation by fluorescence microscopy was used to demonstrate that TNFα stimulated ROS production in a subpopulation (about 27%) of rat hippocampal neurons. The early time course of a responding neuron and a non-responding neuron in the same culture is shown in Figure S2B with effects of peroxide (positive oxidizing control) and N-acetylcysteine (reducing control) also shown. Dissociated hippocampal neurons were infected with Ad-DNp22^PHOX^ or a GFP-expressing adenovirus as a control. Two days post-infection, neurons were treated for 20 h with either Aβd/t (∼250 pM) or TNFα (50 ng/ml), fixed, immunostained for cofilin (Alexa 594 secondary antibody), and infected neurons (GFP positive) scored for rod formation. Aβd/t- and TNFα-treated neurons infected with the control viruses formed rods to the same extent as uninfected cells (included in controls) and were significantly (p<0.001) above untreated neurons. In contrast, rod formation in neurons expressing DNp22^PHOX^ was indistinguishable from untreated controls (Figure 4).

We also tested three different pharmacological inhibitors of NOX for their ability to inhibit rod formation induced by TNFα, Aβd/t or glutamate. TG6-227 inhibits NOX 1 and 2 with an IC_50_ of 200 nM but does not inhibit NOX 3 or 4 (unpublished results from J.D. Lambeth). 2-Acetylphenathiazine (ML171) inhibits NOX1 with an IC_50_ of about 200 nM but has a 10–20 fold higher IC_50_ for NOX2 and 3 [40]. Apocynin is a broad spectrum NOX inhibitor with other off-target effects [41]. When used at 3–10 fold above their IC_50_ value for their most specific NOX isoform target, all of these NOX inhibitors significantly (p<0.001) reduced rod formation to that of untreated controls in both Aβd/t- and TNFα-treated neurons but did not affect rod formation in response to excitotoxic levels of glutamate (Figure 4). Although some of these NOX inhibitors may have other NOX-independent targets [40], taken together with the effects of DNp22^PHOX^, these results strongly suggest TNFα and Aβd/t, but not glutamate, induce rods through a pathway dependent upon NOX activity.

### EGFP-PrP^C^ overexpression is sufficient for rod formation in the absence of exogenous rod-inducers and requires NOX activity

Because prion proteins are linked only via a glycosylphosphatidylinositol (GPI) lipid anchor to the membrane outer leaflet, the mechanism(s) by which they participate in NOX activation and/or signal transduction is not well understood. Since Aβ interacts directly with PrP^C^ [24], and Aβ-induced synaptic damage can be mediated by cross-linking of PrP^C^ [42], we determined if overexpression of PrP^C^, and hence increased density within membrane domains, in itself could be sufficient to induce rod formation. To test this, we infected cultured hippocampal neurons with adenovirus expressing EGFP-PrP^C^ driven by a strong CMV promoter. Results from previous studies using this EGFP-PrP^C^ construct demonstrated that the EGFP-PrP^C^ reached the cell surface [43]. This was confirmed by confocal microscopic inspection of cultured neurons infected with adenovirus for EGFP-PrP^C^ expression, which showed a diffuse membrane labeling (data not shown). Thus the insertion of EGFP did not disrupt PrP^C^ trafficking.

**Figure 5:**
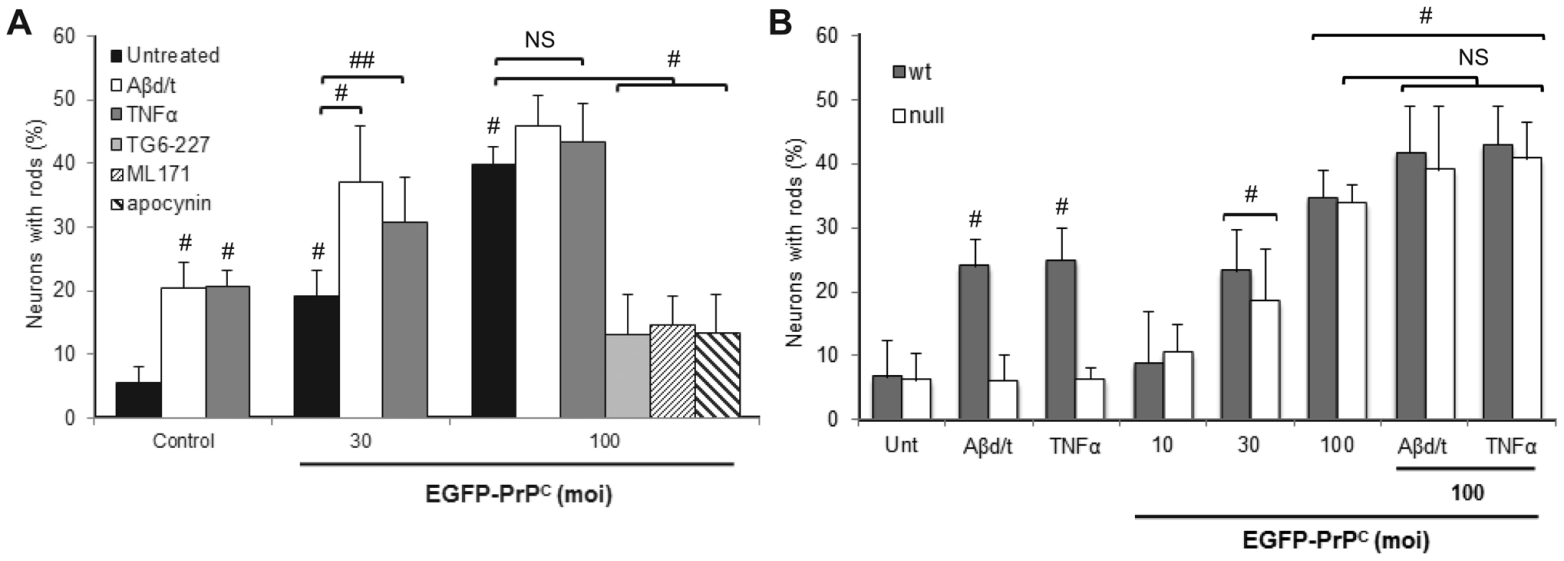
Overexpression of the cellular prion protein induces rods in both rat and mouse neurons and requires NOX for rod formation. (A) E18 rat hippocampal neurons infected with adenovirus expressing EGFP alone (control; 100 MOI) or EGFP-PrP^c^ (30 and 100 MOI) for 60 h were left untreated or treated with Aβd/t or TNFα (20 h) prior to fixing, immunostaining and quantifying the percent of neurons with rods. Neurons infected (100 MOI) with adenovirus for expressing EGFP-PrP^C^ for 60 h were left untreated or treated with the NOX inhibitors at the concentrations used in Figure 4 for 4 h prior to fixation and rod quantification. Within each group, treatments are compared for significance (# p<0.001; ## p<0.005) with respect to the group control (black column). Significance of the EGFP-PrP^C^ infected groups are compared to the untreated control (# p<0.001). (B) P0 hippocampal neurons were obtained from wt and PrP^C^-null FVB pups and plated neurons on day 5 were left untreated or treated with Aβd/t or TNFα as previously described. Some cultures were infected on day 3 with different MOIs of adenovirus for expressing EGFP-PrP^C^ and after 52 h some of the cultures infected with 100 MOI of virus were also treated with Aβd/t or TNFα. All infected cultures were fixed and stained for rods at 72 h after infection (day 6 in culture). PrP^C^-null neurons did not form rods in response to Aβ or TNFα but both wt and PrP^C^-null neurons responded identically by forming spontaneous rods when infected with 30 or 100 MOI of PrP^C^-expressing adenovirus (#p<0.001 from uninfected control).

To determine the consequences of EGFP-PrP^C^ overexpression on rod formation *per se* or in the context of Aβd/t or TNFα, we infected rat hippocampal neurons at different multiplicity of infections (MOI) compared to control adenovirus expressing GFP at the highest MOI. Neurons infected with 100 MOI of adenovirus expressing GFP alone had no increase in rods over untreated controls (included within untreated controls). Of EGFP-PrP^C^ positive neurons infected at an MOI of 30, 19% had rods (p<0.001 compared to control) and this percentage significantly increased when neurons were treated with TNFα (31%, p<0.005) or Aβd/t (37%, p<0.001) (Figure 5A). When neurons were infected with an MOI of 100, 40% of GFP positive cells developed rods (significant at p<0.001 compared to untreated controls) but the slightly increased response to exogenously applied TNFα or Aβd/t was no longer significant (Figure 5A). Therefore, PrP^C^ overexpression alone can induce rods to a level upon which treatment with TNFα or Aβd/t has no significant additional effect.

To determine if neurons from PrP^C^-null mice would form rods upon re-expressing PrP^C^, we compared rod formation between wt and PrP^C^-null hippocampal neurons infected with different MOIs of the EGFP-PrP^C^ adenovirus. Both Aβd/t and TNFα induced rods in the wt but not in the PrP^C^-null neurons (Figure 5B). Spontaneous rod formation occurred to about the same degree in both wt and and PrP^C^-null neurons infected with EGFP-PrP^C^ adenovirus. When infected with an MOI of 10, neurons of either genotype were not significantly different from uninfected controls, but at MOIs of 30 or 100, spontaneous rod formation was significantly (p<0.001) enhanced in wt and PrP^C^-null neurons. Addition of Aβd/t or TNFα to neurons of either genotype infected with 100 MOI did not result in any significant increase in rod formation (Figure 5B). These results demonstrate both the necessity of PrP^C^ for rod formation induced by Aβd/t or TNFα and also the sufficiency of the PrP^C^ overexpression alone for rod induction.

To determine if PrP^C^ expression may have an indirect effect on rod formation through cytokine secretion and a possible autocrine feedback loop, we performed a multiplex assay for 11 cytokines, including all three of the ones tested for rod induction, in medium collected from high density cultures of neurons prepared from PrP^C^-null mice. Some cultures were infected with different MOIs of adenovirus for EGFP-PrP^C^ expression to determine if re-expression of PrP^C^ to levels that induced rods in 40% of the neurons (100 MOI, Figure 5A) also had an impact on cytokine secretion. The sensitivity of the assay was demonstrated to detect each cytokine at least one order of magnitude below the level required to obtain a minimal rod response (1.5 ng/ml in Figure 1). All 11 cytokines in the medium from both uninfected and infected PrP^C^-null neuronal cultures (100 MOI) were below a detectable level. Thus this possible indirect role of PrP^C^-induced cytokine secretion need not be considered further.

We next determined if overexpressed EGFP-PrP^C^ induced rods via a NOX-dependent pathway. Because the DNp22 is expressed from a GFP expressing adenovirus, we could not use this virus for identifying labeled cells in combination with the virus for expressing EGFP-PrP^C^. Therefore we utilized the three NOX inhibitors TG6-227, ML171 and apocynin at the concentrations (3–10 fold higher than their IC_50_) that inhibited rod formation in response to Aβd/t and TNFα (Figure 4). Rod formation in EGFP-PrP^C^ overexpressing rat hippocampal neurons (100 MOI) treated 4 h with any of these inhibitors was significantly (p<0.001) reduced (Figure 5A). Thus, EGFP-PrP^C^ overexpression alone appears to induce rods via the NOX-dependent pathway utilized by Aβd/t and TNFα.

### Treatment of neurons with either Aβd/t or TNFα results in changes in cofilin phosphorylation

**Figure 6:**
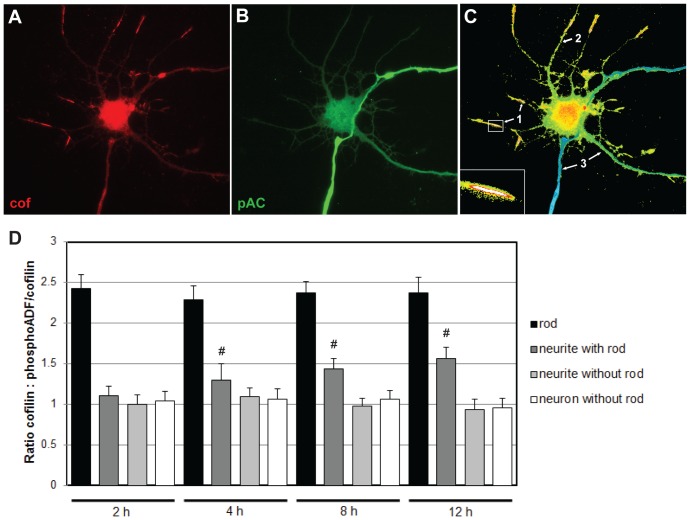
TNFalpha induces cofilin activation within neurites in which rods form but not in other neurites or neurons. (**A**–**C**) Images of a neuron treated with TNFα for 8 h and fixed and immunstained for total cofilin (mouse antibody MAb22) and for phosphorylated ADF/cofilin (rabbit antibody). Example from a single neuron showing (**A**) total cofilin immunostaining; (**B**) phosphorylated ADF/cofilin immunostaining; (**C**) ratio image of total cofilin:phosphoADF/cofilin using a hot scale to show the regions of greatest cofilin activity (red/yellow). Numbers on the figure show the regions used to obtain: 1) ratio directly over rods; 2) ratio in a rod forming neurite but not in the region of the rod; 3) neurites without rods from a neuron with rods in other neurites. (**D**) Quantification of ratio images taken in different regions of neurons fixed at different times (2, 4, 8 and 12 h) after TNFα treatment. We observed a significant (# p<0.001) increase in cofilin dephosphorylation by 4 h after TNFα treatment only in the region of a rod-containing neurite outside of the rod area.

We previously reported that cofilin was rapidly dephosphorylated in neurons treated with agents that induce rods over a short time span, such as glutamate, ATP-depletion medium and peroxide [1]. However, TNFα and other proinflammatory cytokines have been reported to increase cofilin phosphorylation within 4 h in endothelial cells through a RhoA/Rho kinase-dependent pathway [44]. Thus, it is important to know the effects of TNFα on cofilin phosphorylation in neurons. Our prevous work showed that changes in cofilin phosphorylation in Aβ-treated neurons were below those detectable by immunoblotting methods because only 20% of the Aβ-treated neurons form rods and often rods are only within a few processes. Thus we developed an assay based upon immunostaining of phosphorylated ADF/cofilin and total cofilin and used ratio imaging to determine that cofilin dephosphorylation occurred in rod-forming neurites of neurons treated with Aβ oligomer [4]. We applied this same method here to determine if local changes in phosphorylated ADF/cofilin occurred within neurites in which rods formed when neurons were fixed at 2, 4, 8 and 12 h after TNFα treatment. Similar to what was observed for Aβ-treated neurons [4], TNFα treatment led to cofilin dephosphorylation only in rod containing neurites and the dephosphorylation was highest directly over rods (Figure 6 and number 1 in panel C). The only significant (p<0.001) difference observed over the timecourse of TNFα treatment was a gradual increase in dephosphorylated cofilin outside of the rod region but within the neurite containing a rod (Figure 6C number 2 and panel D neurite with rod). Neurites without rods (Figure 6C, number 3 on figure), yet extending from neurons containing rods in other neurites, showed no differences in the ratio image from those measured in non-rod forming neurons. Thus the cofilin dephosphorylation response to both TNFα and Aβd/t is highly localized to the neurites in which rods form.

### Rods induced by the PrP^c^-dependent pathway are dynamic structures and are reversed upon washout of their inducing agent

We previously demonstrated that rods formed in neurons treated for 24 h with Aβd/t disappeared by 24 h after Aβd/t washout without neuronal loss [7]. Here we performed similar studies on rod reversibility after washout of TNFα but limited the exposure to TNFα to 12 h by which time we had reached a maximum rod response. By 12 h after washout of TNFα, or 24 h after washout of Aβd/t, rods were significantly (p<0.001) reduced (Figure 2A). However, to obtain more accurate measurements on the kinetics of rod disappearance we utilized live cell imaging.

In live neurons, the study of cofilin-actin rod formation induced by specific mediators of stress has been limited because overexpression of fluorescent protein-tagged wt cofilin results in formation of considerable numbers of “spontaneous” rods which are exacerbated by the photostress of imaging [3], [45]. We recently reported that a mutated form of cofilin (R21Q)-mRFP serves as a rod reporter in live cells without inducing rods even when expressed at levels 3–5 fold over endogenous cofilin [9]. This reporter incorporated into all rods that formed rapidly in neurons in response to excitotoxic glutamate, but was incorporated only into about half of the rods that formed slowly in response to Aβ and TNFα, suggesting that its weaker actin binding allows it to be excluded from slower forming rods. Nevertheless it can be used as a genetically encoded indicator for studying rod dynamics and disappearance in cultured neurons.

**Figure 7:**
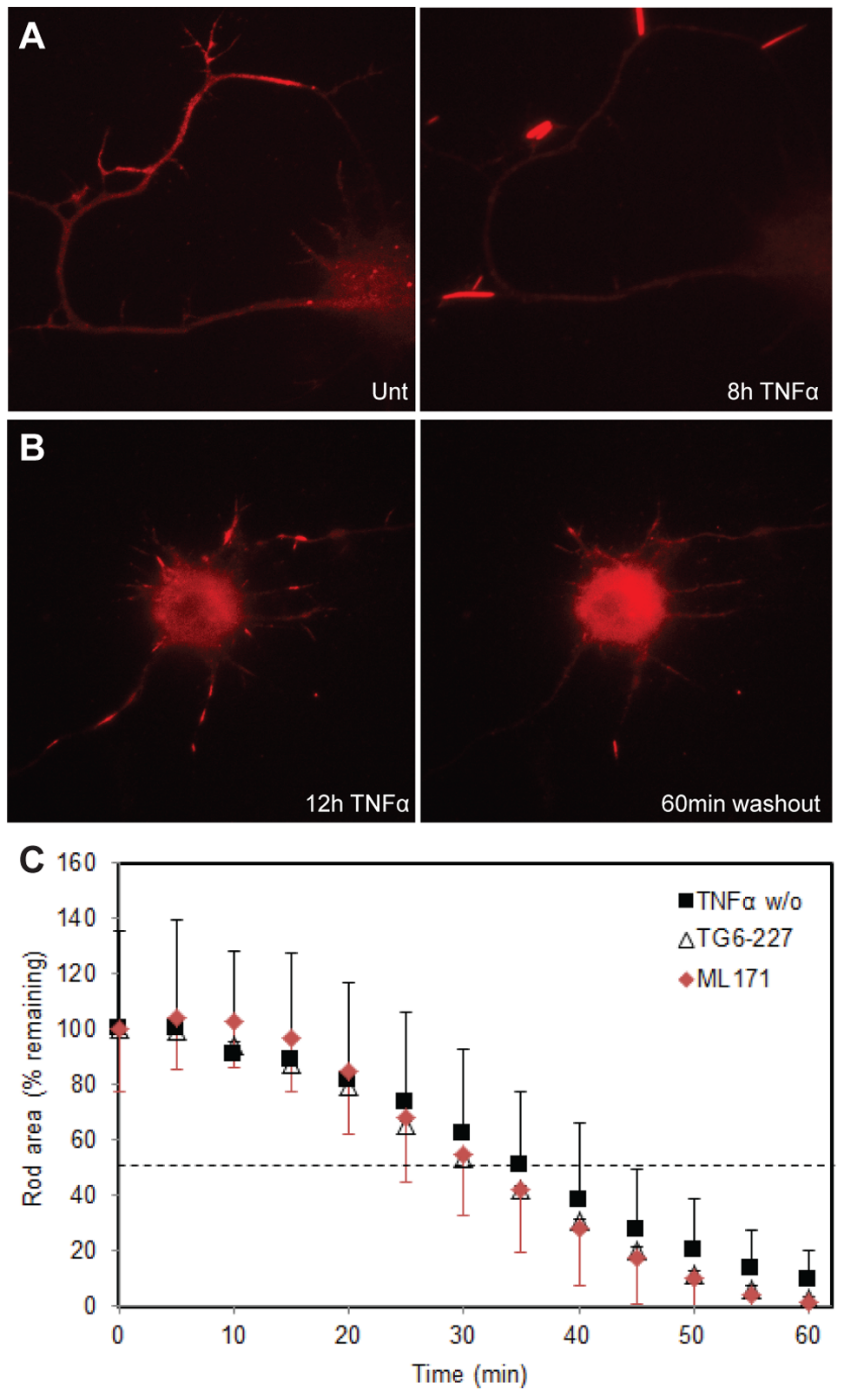
Rod dynamics and rod reversal observed in live neurons. Neurons were infected with adenovirus expressing cofilin(R21Q)-mRFP 48 h prior to imaging. (**A**) Images of neurons untreated (left) or treated with TNFα for 8 h (right). Neurons were imaged at 10 min intervals and dynamics of rods are shown in Movie S1. The rods observed in the right panel showed movement when they were small (<5 µm) but stopped moving when they reached their maximum size (∼10 µm), usually within 20 min of their first appearance. (**B**) Image on left is a neuron treated for 12 h with TNFα in which rods have formed in many processes. Panel on right shows this same neuron 60 min after washout of the TNFα in which almost all rods have disappeared. (**C**) Time course of rod disappearance calculated from measurements on 5 rods per field from 3 independent experiments (15 rods total). Total rod areas were quantified at 5 min intervals for both washout of rod inducer (TNFα w/o) and cultures infected with adenovirus expressing EGFP-PrP^C^ that were treated with TG6-227 or ML171. From this plot a half-life for disappearance of TNFα-induced rods was calculated to be 36 min, which is similar to the half-life of PrP^C^-overexpression-induced rods upon addition of NOX inhibitors (∼31 min). Error bars (std. deviation) for the TG6-227 (not shown) are similar to those shown for the ML171.

Neurons were infected with adenovirus for expressing cofilin(R21Q)-mRFP and, after 36–48 h, treated with Aβd/t or TNFα. Individual neurons were randomly selected for imaging to follow rod formation for several hours. Previously we showed that neurons expressing cofilin(R21Q)-mRFP did not form new rods as a result of photo-stress of imaging [9]. TNFα-induced rod formation is shown in Figure 7A and in Movie S1. No unusual changes were observed in the fluorescence distribution of the cofilin(R21Q)-mRFP prior to the appearance of rods, which elongate to their full length within 20 min once initiated. Rods sequester most of the cofilin within the processes in which they form (compare the diffuse distribution of cofilin between the untreated and 8 h TNFα treatments shown in Figure 7A). Many newly formed rods undergo translocation within the neurite, predominantly in the retrograde direction and they often disassemble and disappear as they near or enter the soma (Figure 7B and Movie S2). Very occasionally small rods were observed undergoing translocation in the anterograde direction. Once rods enlarge to seemingly occlude the neurite, their motility ceases (Figure 7A). Washout of the TNFα resulted in the disappearance of the induced rods with a half life of 36 min (Figure 7C). We also studied the reversal of rods formed in neurons overexpressing PrP^C^. In these neurons, we reversed rod formation by addition of the NOX inhibitors ML171 or TG6-227. The rate of rod reversal with addition of NOX inhibitors is identical to that measured upon washout of the rod inducers (Figure 7C), suggesting that continued NOX signaling through the prion-dependent pathway is required for rod maintenance.

## Discussion

Here we show for the first time that proinflammatory cytokines are able to induce cofilin-actin rods in the same subpopulation of hippocampal neurons that forms rods in response to treatment with Aβ, which were shown to be most highly localized to neurons in and around the dentate gyrus and mossy fiber tract in organotypic cultures of rat hippocampus [46]. Interestingly, the rod formation pathway utilized by the proinflammatory cytokines and Aβ, but not glutamate or energy depletion, requires expression of PrP^C^ and activation of NOX. The activation of NOX produces ROS, which is required for cofilin oxidation and formation of an intermolecular disulfide bond found in cofilin incorporated into rods [16]. We then show EGFP-PrP^C^ overexpression alone drives rod formation through a NOX-dependent pathway, suggesting that peptide/protein inducers of rods signal though PrP^C^-enriched membranes.

Results from several recent studies suggest that Aβ plays an important role in development of cognitive impairment associated with aging and AD [47], [48]. However, levels of Aβ vary widely in extracts of frontal cortex from AD subjects [11] and Aβ profiles overlap considerably with those from cognitively normal subjects of similar ages [49], implying cognitive decline is not simply due to the amount of Aβ produced, but also to its post-production processing [6], [7]. Indeed, extracts of human AD brain prepared using progressively stronger extraction procedures yielded different pools of Aβ; a soluble SDS-stable dimer was among the most synaptotoxic [50] and its levels correlate with dementia in most (but not all) AD subjects [10], [11]. Small SDS-stable Aβ oligomers, including dimers, can be formed by *in vitro* oxidation of synthetic human Aβ under physiologically relevant conditions [51]. Oxidation increases the rod-inducing activity of synthetic human Aβ more than 600 fold [7], almost to the potency obtained with SDS-stable dimer/trimers (Aβd/t) fractionated from the culture medium of Chinese hamster ovary (CHO) cells (line 7PA2) made to secrete human Aβ [52]. Thus, Aβ rod-inducing activity correlates with a major synaptotoxic species extracted from AD brain.

Proinflammatory cytokines have been implicated in the progression of AD, as well as in many other chronic and acute neurodegenerative diseases and certain psychiatric disorders [53]–[56]. In Down's syndrome (trisomy 21), the prevailing cause of mental retardation, there is upregulation of the pluripotent neuroinflammatory cytokine IL-1, which can be brought about by changes occurring as a result of chromosome 21 gene products [56]. Aβ, thought to be an initiator of familial AD, increases proinflammatory cytokine release [19] and proinflammatory cytokines also link traumatic brain injury or epilepsy to later development of Alzheimer-type dementia [57]–[59]. However, there appear to be at least two different cytokine profiles in extracts of frontal cortex from subjects with early AD, one showing a preponderance of proinflammatory cytokines and the other showing elevated anti-inflammatory cytokines [32]. By end stage AD, the phenotypes merge into one showing increases in both pro- and anti-inflammatory markers. Finding different phenotypes in subjects suffering from early AD is not surprising given the multifactorial nature of sporadic AD [60]. However, the different cytokine profiles correlate with significant differences in abundance of extracellular amyloid plaques; lower plaque numbers are found in subjects with elevated pro-inflammatory cytokines [32], suggesting possible differences in the mechanism by which cognitive dysfunction develops in each subject cohort.

The proinflammatory cytokine TNFα enhances ROS production in cells via activation of NOX, reportedly via interaction with its specific cytokine receptor TNFR1 [61], [62]. Polymorphisms in TNFα and in the promoter region of TNFα and IL-6 are associated with increased risk of AD and with late-onset sporadic AD [63]. Although several studies have linked Aβ to prion-dependent neurodegenerative mechanisms involving membrane microdomains (lipid rafts) [64]–[66], our results are the first to demonstrate a requirement for the cellular prion protein in linking TNFα signaling to NOX activation in neurons. This finding suggests proinflammatory cytokines working through their specific receptors may use a PrP^C^-mediated signaling pathway and that many promiscuous but high affinity Aβ binding partners, such as LilrB2 (PirB in mouse) [14] or mGluR5 [26], also may be linked via prion-dependent pathways to neurodegeneration. Such a linkage was shown for Aβ interacting with the metabotropic glutamate receptor mGluR5; PrP^C^ is required as a co-receptor to activate the non-receptor tyrosine kinase fyn [26], [28], as well as for the cognitive dysfunction in mice overproducing Aβ [33]. Furthermore, disruption of membrane microdomains by ganglioside depletion protected hippocampal neurons from toxicity by an amyloid form of calcitonin [64], suggesting that PrP^C^-involvement through membrane microdomains may be a common denominator for amyloid-induced neurotoxicity. Indeed, mice expressing GPI anchorless PrP^C^ outside of microdomains do not develop clinical prion neuropathology despite accumulating high titers of infectious amyloid [67]. These findings suggest that many amyloid/prion-associated neurodegenerative diseases may have a common mechanism through altered prion-signal transduction [68]

Two competing outcomes for cofilin sulfhydryl oxidation have been characterized [69]. Cofilin sequestered in rods contains one intermolecular disulfide [16]. However, cofilin can undergo oxidation to form two intramolecular disulfide bonds, eliminating its actin binding but targeting it to mitochondria where it mediates apoptosis via cytochrome c release [70]. Aβ-induced neuronal apoptosis depends upon mitochondrial targeting of cofilin [71] and requires the scaffolding protein RanBP9, a mediator of cofilin activation and ROS production [15]. Therefore, although rods block transport and cause synaptic dysfunction [3], [9], [69], they also spare ATP used in actin turnover [45] and may promote neuronal survival by preventing complete cofilin oxidation and thus its mitochondrial targeting. AD is characterized in early stages as a disease of synapses and connectivity, not of neuronal death, which occurs later in disease progression [72]. How frequently and over what extended period rods may form and disappear during the early stages of AD is a question that needs to be addressed to determine if rods have some beneficial effects in neuronal survival.

Rod formation requires both the activation of cofilin (dephosphorylation) and its oxidation. These two events may be regulated in parallel or independently. Neuronal rod formation in response to Aβ is slow (about 12 hr to maximum response) and both the percentage of responding neurons and the numbers of rods formed can be greatly suppressed by overexpressing activated LIMK1, a cofilin kinase [7]. However, in neurons treated with glutamate or mitochondrial poisons, rods form rapidly (within 30 min) and in almost every neurite; cofilin dephosphorylation also occurs rapidly within this timeframe [1] as ATP levels drop and the cofilin phosphatase chronophin is released from complex with hsp90 [73]. Mitochondrial production of ROS is also rapid [74]. One pathway that could mediate both cofilin dephosphorylation and oxidation utilizes the scaffolding protein 14-3-3ζ, which binds both to phospho cofilin [75] and to the cofilin phosphatase slingshot (SSH1-L), keeping them inactive [76]. 14-3-3ζ contains two cysteines that are oxidized to form a disulfide upon ROS exposure, releasing it from SSH1-L and allowing phosphatase activation and cofilin dephosphorylation in an oxidative environment [77].

Virtually all neurons are capable of forming rods when energy depleted and thus the question arises as to why Aβ and proinflammatory cytokines induce rods in only 20–25% of neurons, about the same percentage of neurons (27%) that showed increased ROS production in response to TNFα. Although PrP^C^ is widely expressed in neurons, there are certain neurons in which its expression is below levels of detection, either by immunostaining [78], [79] or through direct visualization in transgenic mice expressing PrP^C^-EGFP [80]. Furthermore, PrP^C^ levels are much lower in dendrites of some neuronal populations than in their axons. Thus, it is not surprising that there is a differential neuronal response to rod formation via the prion-dependent pathway and that increased expression of EGFP-PrP^C^ in either wt or PrP^C^-null neurons increases their rod response in a NOX-dependent manner. The PRNP gene that encodes PrP^C^ contains polymorphisms at codon 129 (met/val), a known susceptibility factor for Creutzfelt-Jakob disease [81], . A comprehensive meta-analysis of M/V polymorphism revealed a modest but significant association with a decreased risk for AD, which is of interest because the polymorphism occurs nearby the resides (92–110) implicated in binding to Aβ oligomers [83]. However, it was surprising to find that overexpression of PrP^C^ in the absence of additional treatment is sufficient to induce rods in 40% of hippocampal neurons. Because PrP^C^-crosslinking mediates synaptic damage [42] and neuronal death *in vivo* [84], we hypothesize that the increased amount of PrP^C^ promotes formation of enlarged membrane domains within which NOX accumulates and becomes activated by other domain components to generate ROS above the threshold required for cofilin oxidation and rod formation. Potential components for NOX activation that associate with the prion-enriched membrane domains are caveolin-1, a recruiter of the non-receptor tyrosine kinase fyn, which may directly or indirectly participate in NOX activation [68], [85]. Such a model for rod signaling provides a potential mechanism by which altered cholesterol homeostasis and stabilization of the enlarged PrP^C^-containing membrane domains might contribute to Alzheimer disease pathogenesis [86].

The rapid reversal of rods following removal of the TNFα (Figure 7C) or Aβd/t [9], or following the addition of NOX inhibitors to neurons overexpressing EGFP-PrP^C^ (Figure 7C), suggests that rod maintenance requires continued ROS production. However, we cannot rule out that PrP^C^ overexpression drives its mislocalization, for example from axons into dendrites, and that increased rod formation occurs as a result of ROS production in a cellular domain in which PrP^C^ levels are normally quite low. Nevertheless, live imaging of rod formation, transport and disappearance using the reversible rod reporter cofilin-R21Q-mRFP supports the idea that rods formed in neurites in response to Aβ or TNFα might initially be transient at sites in which a ROS threshold is exceeded for a short time, and only some fraction of these remain stable enough to grow into more persistent rods.

The activation of a specific NOX isoform depends upon recruitment of specific cytoplasmic subunits. NOX1-4 associate with the stabilizing membrane protein p22^PHOX^ [40]. NOX1 is strongly inhibited by ML171, whereas NOX1 and 2 are inhibited by TG6-227 and all are inhibited by apocynin. Thus it is likely NOX1 and NOX2 are the major isoforms involved in Aβ and TNFα-induced ROS production. Subunit recruitment for NOX2 in response to Aβ oligomers is dependent upon the activation of phospholipaseA_2_α (cPLA2) and neutral sphingomyelinase to generate ceramide [87], [88]. Future directions will determine if overexpressing PrP^C^ activates cPLA2, generates ceramide, if the site of this activation defines the position of rods, and if the duration and intensity of NOX activation dictates rod persistence.

The vast majority of AD cases are considered sporadic in incidence and multifactorial in cause, making treatment of the disease at an early stage challenging [89]. Thus, being able to bridge multiple disease initiating mechanisms, such as Aβ overproduction or neuroinflammation triggered by proinflammatory cytokines, into a common pathway leading to synapse loss provides an attractive focus for therapeutic agents. The formation of cofilin-actin rods provides such a target.

## Materials and Methods

### Ethics Statement

All animals were handled according to National Research Council's Guidelines to Care and Use of Laboratory Animals as approved by the Colorado State University Institutional Animal Care and Use Committee (approved protocol #11-3951A).

### Reagents

All chemical reagents are from Sigma-Aldrich Co. (St. Louis, MO), and all tissue culture and fluorescence reagents are from Life Technologies (Invitrogen Corp., Carlsbad, CA) unless otherwise indicated.

### Neuronal Cell Culture

Rat E18 cortical and hippocampal neurons were obtained from timed-pregnant dams (Harlan, Indianapolis, IN) and used fresh or stored frozen as previously described [1]. Cells (15-20,000) were plated on poly-D-lysine-coated coverslips (Glaswarenfabrik Karl Hecht KG, Sondheim, Germany) either in drilled out 35 mm tissue culture dishes (22 mm square coverlips attached with aquarium sealant) or in 24 well plates (15 mm round cover slips) and cultured in Neurobasal SFM (serum free medium) (GIBCO, Grand Island, NY), supplemented with B-27 (used at 1X; Life Technologies), GlutaMax (25 µl/10 ml; Life Technolgies) and Penicillin/Streptomycin (50 units/ml/50 µg/ml final concentration). Unless otherwise indicated, experiments were performed with E18 rat hippocampal neurons. Mouse neurons were obtained from two wild type (C57BL/6, FVB) lines and from the PrP^C^-null FVB line. For the mouse cell cultures, hippocampi were removed from newborn pups (P0) and were cultured as described for the rat neurons except medium was supplemented with 25 µM 2-mercaptoethanol and 25 µM glutamate for the first 3–4 days in culture to help reduce spontaneous rod formation. Cultures were maintained in a 5% CO_2_ incubator at 37°C.

### Adenovirus Preparation

Adenoviruses were made using the AdEasy system [39] modified as previously described [90]. pRedTrackCMV was prepared by removing the GFP cDNA from pAdTrackCMV [39] with Agel and Bcll. The mRFP cDNA [91] was amplified with a 5′ primer containing an Xmal site and a 3′ primer containing a BglII site. Using compatible cohesive ends involving a three part ligation annealing, the mRFP was ligated into pAdTrackCMV in place of the GFP cDNA. pAdTrackCMV and pRedTrackCMV were used for making virus for expressing a dominant negative form of the NOX subunit p22^PHOX^ (DNp22^PHOX^). Briefly, the coding region of DNp22^PHOX^ cDNA [37] was excised with SpeI/BclI and ligated into pAdTrackCMV or pRedTrackCMV cut with XbaI/BglII. Plasmids were purified and digested with PmeI prior to electroporation into BJ5183 *E. Coli* cells containing the pAdEasy-1 vector. Adenoviruses for expressing DNp22^PHOX^ or GFP (the pAdTrack CMV used directly) were then made and titered [90].

pShuttle CMV [39] was used to make adenovirus for expression of lacZ-GFP or mRFP (for control infection), cofilin-R21Q-mRFP, and EGFP-PrP^C^ or EGFP-linked via a GPI group to membrane. The EGFP-PrP^C^ coding region was cut from the wt plasmid containing mouse PrP^C^ in the pEGFP-C1 vector [43]. The EGFP-GPI control came from the same plasmid with the PrP^C^-coding region removed. Briefly, the coding regions were excised with NheI/SmaI, and ligated into the XbaI/EcoRV sites of pShuttleCMV. Adenoviruses for expressing the proteins were then made and titered [90]. Adenoviruses for expression of cofilin (wt)-mRFP and cofilin(R21Q)-mRFP in pShuttle behind a neuronal specific enolase promoter, cofilin promoter or CMV promoter have been described [9].

### Characterization of Adenoviruses

All new adenoviruses were tested for expression of the encoded protein by infection of either SAOS2 or N2a cells, waiting until fluorescence was readily visible in at least 50% of the cells (usually by 36 h postinfection) and then lysing the washed cells in an SDS-lysis buffer [92] and performing Western blots using antibodies against the protein of interest and GAPDH as an internal standard for normalization. Chimeric proteins with EGFP or mRFP had migration mobility equivalent to a mass of about 25 kDa above the endogenous non-chimeric protein. Experiments were usually performed 48–72 h after infection for expressing fluorescent proteins either as chimeras or infection markers.

### Cell Treatments

#### Adenovirus infection

Neurons were cultured for 2–3 d before infection at 30–300 multiplicity-of-infection (MOI) with adenoviruses for expressing different proteins. Unless otherwise stated, experiments were performed 3 d post-infection. Infection was performed by removing 0.5 ml of the medium, mixing it with virus and adding it back to the well.

#### Rod inducing treatments

For rod induction cells were treated with TNFα, IL-1β, or IL-6, dissolved in neurobasal medium at 100x the final concentration used in culture. Aβ dimer/trimer (Aβd/t) was fractionated from the 10× concentrated culture medium of 7PA2 cells [93] on a Superose-75 gel filtration column. Fractions containing the majority of the SDS-stable Aβ dimer and trimer were identified by Western blotting [52], combined, and freeze dried. Immediately before use, the Aβd/t was dissolved in complete medium to its original secreted concentration (1X) [7] and used to replace the medium on the neurons. Rods were also induced by medium addition for 30–60 min to a final concentration of 2 µM antimycin A or 150 µM glutamate, or by incubation of neurons in PBS containing 10 mM Na azide/6 mM 2-deoxy-D-glucose (ATP-depletion). NOX inhibitor TG6-227 was dissolved in DMSO at 100 µM and used at 1 µM in culture. ML171 was dissolved in DMSO at 100 µM and used at 500 nM and apocynin was dissolved in DMSO at 1 mM and used at 1 µM in culture. The usual vehicle control was DMSO at 0.1% final concentration but in some experiments DMSO was used at 1% without detrimental effects over 12 h.

### Fluorescent Labeling of Rods

For rod quantification, cells were fixed in 4% formaldehyde, 0.1% glutaraldehyde in PBS for 45 min at room temperature, permeabilized with methanol (chilled at −20°C) for 3 min, and blocked with 5% goat serum in 1% bovine serum albumin/Tris-buffered saline before cofilin immunolabeling with affinity purified rabbit 1439 antibody (2 ng/µl) [94] and fluorescent secondary antibodies. Coverslips were mounted with ProLong Gold Antifade (Invitrogen). For ratio imaging of phosphoADF/cofilin versus total cofilin, rabbit 4321 phospho ADF/cofilin antibody (affinity purified at 1 ng/µl) and MAb22 [95] (total IgG at 2 ng/µl) against total cofilin were the primary antibodies used. Secondary goat anti-rabbit antibodies (1∶450 dilution) were labeled with Alexa 488, Alexa 594 or Alexa 648.

### Fluorescence Microscopy and Image Analysis

Images were obtained from fixed dissociated neurons on an inverted Nikon Diaphot microscope with a CoolSnap ES camera controlled by Metamorph software. Scoring for rods was performed blindly; randomized samples were not identified until all coverslips had been scored. Coded coverslips were scanned over several different regions and for most experiments100 neurons per coverslip were examined and scored as positive for rods if they contained a single rod. Rod-containing neurons interacting with other neurons were scored as one positive neuron since it was not possible to determine from which soma a rod containing process originated, whereas non-rod-containing neuronal networks were scored for each soma that they contained since none of the neurons within the network had rods. Triplicate or quadruplicate coverslips for each treatment were used in each experiment and experiments were repeated at least three times, giving between 800 and 1200 neurons scored for each treatment.

Live cell imaging was performed on a Nikon Eclipse 2000 inverted TIRF microscope with 405, 488, 561 and 640 nm laser lines, perfect focus control, XY piezo Z stage, CO_2_-controlled stage incubator, 100X (1.48 NA) and 40X (0.75 NA) objectives and Andor iXon3 EMCCD camera. Images were captured and analyzed using Nikon Elements software.

### Statistics

Unless otherwise stated, all experiments for which quantitative data is provided were performed with at least triplicate samples and were repeated at least three times. Statistical significance between samples with one variable was calculated using Student's “*t”* test, whereas significance of differences between groups with multiple variables was performed by ANOVA with Tukey's post-hoc analysis using JMP software (SAS Institute Inc.). Statistical comparisons in the figures utilize the following symbols for the p values given: * p<0.01, ** p<0.05, # p<0.001, and ## p<0.005. NS  =  not significant.

## Supporting Information

Figure S1 **Brain expression levels of NOX1 and NOX2 are similar in wild type and PrP^C^-null mice. (A)** Typical western blot of extracts from the cortex of two wt and two PrP^C^-null FVB mice showing bands for NOX1 and GAPDH. **(B)** Quantitative information from duplicate blots of duplicate extracts in which intensities of NOX1 and NOX 2 bands were normalized to GAPDH. There are no significant differences of in the brain expression levels of NOX1 and NOX2 between wt and PrP^C^ null mice. Bars  =  std. deviation.
(TIF)

Figure S2 **Measurements of reactive oxygen species (ROS) using the DCF assay. (A)** SAOS2 cells, an osteosarcoma cell line that infects very efficiently with low levels of adenovirus, were kept uninfected (Ctrl), or infected with a control adenovirus (VirCtrl) or with adenovirus for expressing DNg22^PHOX^. After 48 h, cells were loaded with DCF-diacetate (20 µM) for one hour, washed, and then left untreated or treated with phorbol myristate acetate (PMA; 400 ng/ml) or peroxide (500 µM) for 30 min before lysis and quantification of lysate for fluorescence and protein. Results displayed show relative fluorescence per mg/ml of total soluble protein to correct for differences in cell numbers per well and all values were normalized to controls. Infection with control virus had no effect on the ability of the cells to generate a ROS response to PMA but expression of DNp22^PHOX^ inhibited the response. The peroxide positive control shows the maximum changes that could be detected in this assay. Results are from quadruplicate samples from a single experiment with error bars showing standard deviation. **(B)** Changes in intracellular DCF fluorescence measured over the soma of two neurons 5 min before and at 10–30 sec intervals for10 min after treatment with 100 ng/ml TNFα. Average intensity per unit area is normalized to pretreatment values at 0 time. In multiple experiments (n = 9) using either 100 ng/ml of 50 ng/ml TNFα, 19 out of 69 (27%) cells imaged over time showed a DCF fluorescence response similar to the responding cell and the other 50 showed no response (labeled here as control). This responding population is not significantly different from the 20–25% of neurons that formed rods in response to 50–100 ng/ml TNFα shown in Figure 1A. After 10 min, peroxide was added to 500 µM to demonstrate a positive response in every cell and about 5 min later excess reducing agent (1 mM N-acetylcysteine; NAC) was added to reverse the oxidative response.
(TIF)

Movie S1 **Dynamic rods precede TNFα-induced stable rods along neurites.** Neurons infected 48 h with adenovirus expressing cofilin(R21Q)-mRFP were treated with TNFα and imaged every 10 min for 8 h immediately following treatment. Rods appeared within 2 hours but did not become stationary until ∼6 hours at which time they appeared to occlude the neurite.
(AVI)

Movie S2 **Reversal of TNFα-induced rods occurs rapidly upon washout of TNFα.** Neurons were infected 48 h with adenovirus expressing cofilin(R21Q)-mRFP and treated with TNFα for 12 h. Medium was removed and replaced with fresh medium without TNFα. Images were taken every 2 min for 1 h. Rod disassembly often was accompanied by their retrograde transport, often segmenting into several smaller rods before finally disappearing close to the soma.
(AVI)

Text S1 (DOCX)
