# Supplementary figures and images for: Amyloid-β and Proinflammatory Cytokines Utilize a Prion Protein-Dependent Pathway to Activate NADPH Oxidase and Induce Cofilin-Actin Rods in Hippocampal Neurons
Source: PLoS One. 2014 Apr 23;9(4):e95995. doi: 10.1371/journal.pone.0095995 (PMC3997518; doi:10.1371/journal.pone.0095995)

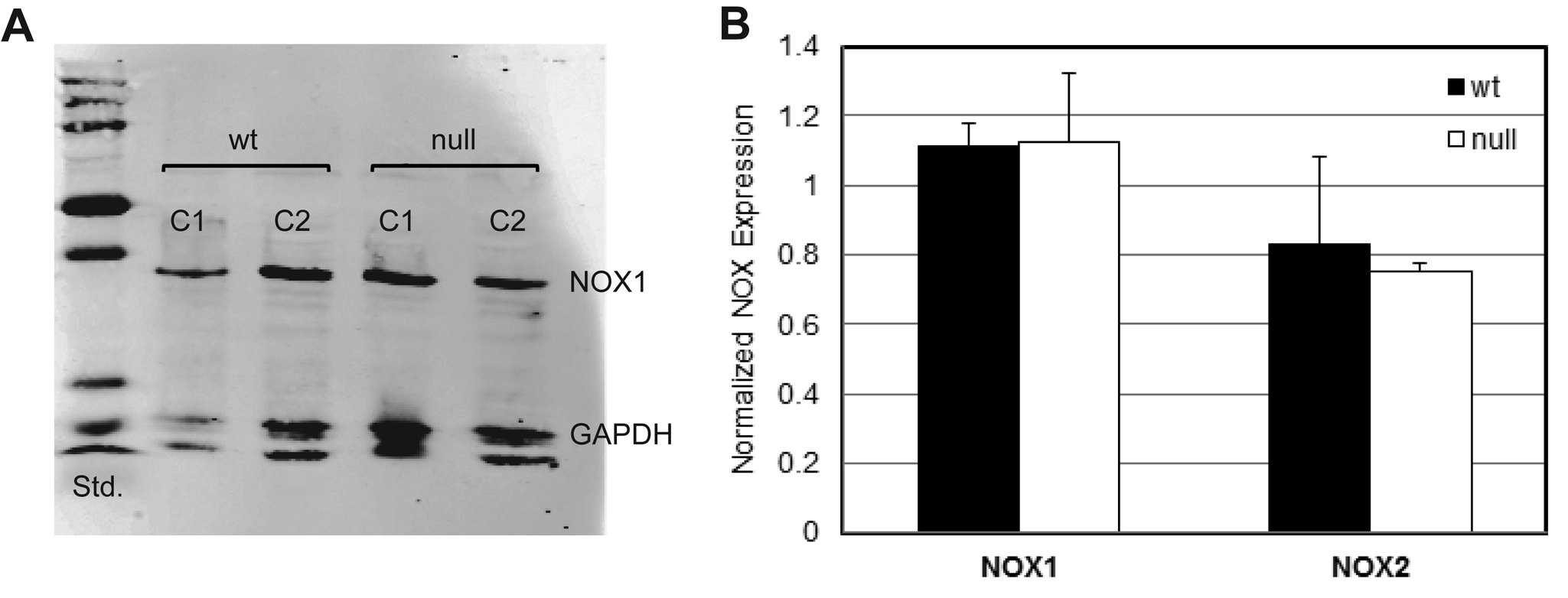

Supplement: Figure S1 — Brain expression levels of NOX1 and NOX2 are similar in wild type and PrPC-null mice. (A) Typical western blot of extracts from the cortex of two wt and two PrPC-null FVB mice showing bands for NOX1 and GAPDH. (B) Quantitative information from duplicate blots of duplicate extracts in which intensities of NOX1 and NOX 2 bands were normalized to GAPDH. There are no significant differences of in the brain expression levels of NOX1 and NOX2 between wt and PrPC null mice. Bars = std. deviation. (TIF) [file pone.0095995.s001.tif]

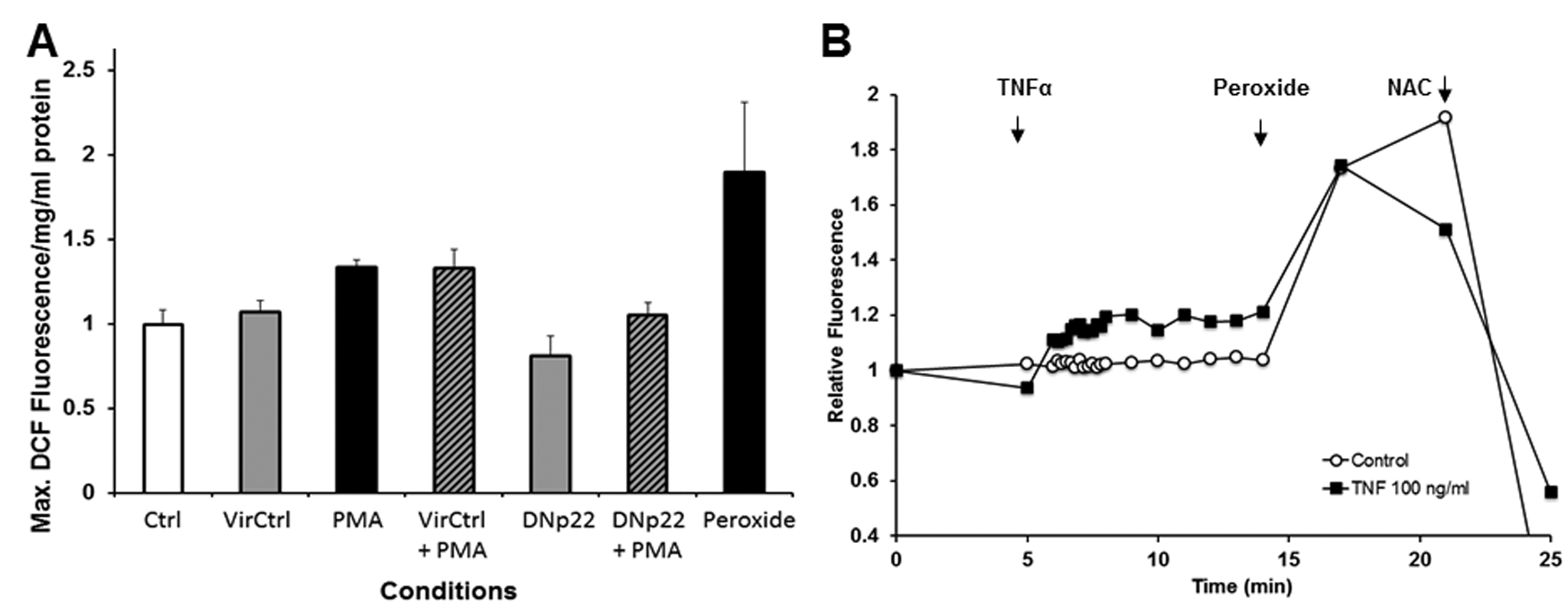

Supplement: Figure S2 — Measurements of reactive oxygen species (ROS) using the DCF assay. (A) SAOS2 cells, an osteosarcoma cell line that infects very efficiently with low levels of adenovirus, were kept uninfected (Ctrl), or infected with a control adenovirus (VirCtrl) or with adenovirus for expressing DNg22PHOX. After 48 h, cells were loaded with DCF-diacetate (20 µM) for one hour, washed, and then left untreated or treated with phorbol myristate acetate (PMA; 400 ng/ml) or peroxide (500 µM) for 30 min before lysis and quantification of lysate for fluorescence and protein. Results displayed show relative fluorescence per mg/ml of total soluble protein to correct for differences in cell numbers per well and all values were normalized to controls. Infection with control virus had no effect on the ability of the cells to generate a ROS response to PMA but expression of DNp22PHOX inhibited the response. The peroxide positive control shows the maximum changes that could be detected in this assay. Results are from quadruplicate samples from a single experiment with error bars showing standard deviation. (B) Changes in intracellular DCF fluorescence measured over the soma of two neurons 5 min before and at 10–30 sec intervals for10 min after treatment with 100 ng/ml TNFα. Average intensity per unit area is normalized to pretreatment values at 0 time. In multiple experiments (n = 9) using either 100 ng/ml of 50 ng/ml TNFα, 19 out of 69 (27%) cells imaged over time showed a DCF fluorescence response similar to the responding cell and the other 50 showed no response (labeled here as control). This responding population is not significantly different from the 20–25% of neurons that formed rods in response to 50–100 ng/ml TNFα shown in Figure 1A. After 10 min, peroxide was added to 500 µM to demonstrate a positive response in every cell and about 5 min later excess reducing agent (1 mM N-acetylcysteine; NAC) was added to reverse the oxidative response. (TIF) [file pone.0095995.s002.tif]
