# Supplementary material for: Amyloid-β and Proinflammatory Cytokines Utilize a Prion Protein-Dependent Pathway to Activate NADPH Oxidase and Induce Cofilin-Actin Rods in Hippocampal Neurons
Source: PLoS One. 2014 Apr 23;9(4):e95995. doi: 10.1371/journal.pone.0095995 (PMC3997518; doi:10.1371/journal.pone.0095995)
Supplement: Text S1 — (DOCX) [file pone.0095995.s005.docx]

Supporting Information for

Amyloid-β and proinflammatory cytokines utilize a prion protein-dependent pathway to activate NADPH oxidase and induce cofilin-actin rods in hippocampal neurons.

K. P. Walsh, L. S. Minamide, S. J. Kane, A. E. Shaw, D. R. Brown, B. Pulford, M.D. Zabel, J. D. Lambeth, T. B. Kuhn, J. R. Bamburg

**Supplemental Methods**

**Protein Assay:** Proteins were precipitated from SDS-lysis buffer extracts of cells and tissues using methanol/CHCl_3_ [1], re-suspended in SDS-PAGE sample loading buffer [2] and protein concentration determined by a filter paper dye-binding assay [3].

**Western Blots:** Proteins separated by SDS-PAGE on 12% isocratic polyacrylamide gels were transferred to nitrocellulase membrane, blocked and immunostained for NOX1 (Boster Biol. Techn. Co antibody PA1666 used at 500 ng/ml), NOX2 (BIOSS antibody bs-3889R used at 2ng/ml) and GAPDH (Millipore antibody MAB374 used at 167ng/ml). Secondary antibodies were labeled with DyLight (ThermoFischer) and blots were scanned using an Odyssey IR scanner (LiCor Instruments). The digitized image bands were quantified using ImageQuantTL software (GE Healthcare).

**DCF Fluorescence Assay:** The assays to measure DCF fluorescence produced by ROS in intact cells and in cell lysates have been described elsewhere [4].

**Supplemental References**

1. Wessel D, Flügge UI. (1984) A method for the quantitative recovery of protein in dilute solution in the presence of detergents and lipids*.* Anal Biochem 138: 141-143.

2. Laemmli UK. (1970) Cleavage of structural proteins during the assembly of the head of bacteriophage T4. Nature 227: 680-685.

3. Minamide LS, Bamburg JR. (1990) A filter paper dye-binding assay for quantitative determination of protein without interference from reducing agents or detergents. Anal Biochem 190: 66-70.

4. Kuhn TB. (2014) Oxygen radicals elicit paralysis and collapse of spinal neuron growth cones upon exposure to proinflammatory cytokines. Biomed Res Int (in press)
